# Suspected undiagnosed ADRD among Middle Eastern and North African Americans

**DOI:** 10.21203/rs.3.rs-1983254/v1

**Published:** 2023-03-24

**Authors:** Tiffany Billmeier Kindratt, Kristine J Ajrouch, Laura B Zahodne, Florence J. Dallo

**Affiliations:** University of Texas at Arlington; U-M: University of Michigan; U-M: University of Michigan; Oakland University

**Keywords:** Middle Eastern and North African, Arab American, Alzheimer’s disease and related dementias, clinical diagnosis, underdiagnosis, National Health Interview Survey, Medical Expenditure Panel Survey

## Abstract

**Background:**

ADRD underdiagnosis among minority populations is well-established and known to be more prevalent among women. Yet, it remains unclear if these patterns exist among Middle Eastern and North African (MENA) adults. We estimated ADRD underdiagnosis among MENA and other US- and foreign-born non-Hispanic Whites and compared sex-stratified results.

**Methods:**

We linked 2000–2017 National Health Interview Survey and 2001 –2018 Medical Expenditure Panel Survey data (ages > = 65 years, n = 23,981). Undiagnosed ADRD was suspected if participants reported cognitive limitations without corresponding ADRD diagnosis.

**Results:**

Undiagnosed ADRD was highest among MENA adults (15.8%) compared to non-Hispanic Whites (US-born = 8.1%; foreign-born = 11.8%). MENA women had 2.52 times greater odds (95% CI = 1.31 –4.84) of undiagnosed ADRD compared to US-born White women after adjusting for risk factors.

**Discussion:**

This study contributes the first national estimates of undiagnosed ADRD among MENA adults. Continued research is needed to facilitate policy changes that more comprehensively address health disparities and related resource allocation.

## Introduction

The prevalence of Alzheimer’s disease and related dementias (ADRD) is increasing worldwide, with 6.5 million adults living with ADRD in the United States (US) (1). This figure is likely an underestimate due to lack of clinical diagnosis. Previous research suggests only half of individuals with ADRD have been diagnosed (2). Early diagnosis and awareness can be important for mitigating safety risks, preventing and treating complications, improving quality of life, and planning for future care (2).

Several studies have established disparities in ADRD incidence, prevalence, and underdiagnosis among minority populations (1–4). The National Institutes of Health recognizes American Indian/Alaskan Native, Asian, Black/African American, and Native Hawaiian or Other Pacific Islanders for race and Hispanic/Latinx for ethnicity as the minimum required categories for reporting health estimates based on Office of Management and Budget (OMB) guidelines (5). Studies have demonstrated that non-Hispanic Black and Hispanic individuals are more likely to have ADRD and be undiagnosed than non-Hispanic Whites (hereafter referred as Whites) (2–4). Fewer studies have examined ADRD disparities among Middle Eastern and North African (MENA) adults, who are included in the White classification by OMB federal guidelines despite a growing body of research suggesting MENA health differs significantly from Whites (6).

The MENA population includes Arab and non-Arab individuals who were born in or trace their ancestry to countries located in the Middle East or North Africa. The Arab population in the US grew by 72% to over 3.7 million between 2000 and 2010 (7). Data from the American Community Survey estimates that 8% of older adults (ages 65 + years) are MENA, which is greater than several other represented minority populations, including Pacific Islanders (5.8%) and Hispanics (5.5%) (8, 9). Despite representing an increasing proportion of the aging population, MENA adults are often underrepresented in studies on cognitive aging because they are difficult to identify in national datasets. Few studies, therefore, have examined the national prevalence of ADRD among MENA adults, regardless of diagnosis. Two studies found that MENA adults were more likely to report cognitive limitations, a potential indicator of ADRD, when compared to US-born Whites (10, 11). Other research demonstrated foreign-born MENA adults had higher estimates of cognitive limitations compared to US-born MENA adults (12). The US prevalence of undiagnosed ADRD among MENA adults remains unknown.

The lack of ethnic identifier to recognize MENA populations contributes to an additional layer of underrepresentation overlooking the health of MENA women. ADRD is known to be more prevalent among women in general, yet it remains unclear if this pattern exists among MENA women. Studies on the physical health of MENA adults in the US have highlighted how MENA men report better health outcomes than MENA women regardless of immigrant status (13). In a study evaluating hypertension and self-reported health among foreign-born MENA, foreign-born Mexican, and US-born Whites, Read & Reynolds found that 19.63% of MENA women reported having hypertension compared to 13.03% of MENA men (13). Despite reporting higher use of healthcare (74% MENA women sought care in last 12 months compared to 58.38% MENA men) and a usual place for care, 17.45% of MENA women self-reported fair/poor health, compared to 9.07% of MENA men, and higher than both foreign-born Mexican and US-born White men and women. How sex shapes the observed differences of ADRD at the intersection of nativity status remains unknown among MENA adults.

To fill this gap in the literature, our aims were to: 1) calculate and compare estimates of suspected undiagnosed ADRD among foreign-born MENA adults compared to US- and foreign-born Whites and 2) compare estimates stratified by sex.

## Methods

### Data Sources

The sample was created by linking 2000–2017 NHIS person level and 2001–2018 MEPS household and medical condition files. Details of both surveys are provided elsewhere (14, 15). Briefly, the NHIS collects individual health information from a sample of households every year. A subsample of participants is selected to participate in the MEPS the following year. The MEPS collects additional information on current medical conditions and health care visits in the last 12 months. Medical conditions provided to interviewers by participants are coded based on the International Classification of Disease (ICD) diagnostic guidance by professional coders and included in specific medical condition files. From 2001 – 2015, ICD-9 codes were created. From 2016–2018, ICD-10 codes were created. To ensure confidentiality, only the first three digits of diagnostic codes were reported in public-use files. Household and medical condition MEPS files were merged with NHIS person files. More information has been reported previously (10).

### Inclusion and Exclusion Criteria

The sample was limited to White adults ages 65 + years (n = 24,827). Adults whose MEPS medical condition file included any ICD-9 (290/294/331/797) or ICD-10 (F01/F03/G30/G31) codes indicating ADRD (16–18) were excluded (n = 846). The final sample included 23,069 US-born White adults, 774 foreign-born Whites from Europe and 138 foreign-born MENA adults.

### Measures

#### Independent Variable

The independent variable was created using the region of birth variable from NHIS. The NHIS includes two questions of country of birth. Participants are asked whether they were born in the US or a US territory overseas. Foreign-born participants were asked their country of birth. To protect confidentially, the NHIS provides a region of birth variable which combines responses into 10 world regions, including the US, Europe, Russia/former USSR countries, Middle East, and Africa, among others. Two foreign-born White groups were created. Responses from White adults born in Europe and Russia/former USSR countries were combined (hereafter, referred to as Europe). Responses from White adults born in the Middle East and Africa were combined based on previous research (10). By including foreign-born White Africans, our study aligns with 2015 US Census content testing for race and ethnicity (6, 19) currently proposed for the 2030 Census (20). The final independent variable included US-born Whites, foreign-born Whites from Europe and foreign-born MENA adults.

#### Dependent Variable

The dependent variable (suspected undiagnosed ADRD) was created using the cognitive limitation variable from MEPS. Undiagnosed ADRD was suspected if a participant reported having a cognitive limitation but did not have a corresponding ADRD diagnosis. The MEPS classifies participants as having any cognitive limitation (0 = no; 1 = yes) if they self-report (or reported by proxy) having problems making decisions, requires supervisions for their own safety, or experiences confusion or memory loss.

#### Covariates

Covariates were selected based on risk factors for cognitive limitations and diagnosis of ADRD identified in previous studies (10, 21). Demographic variables examined were age (continuous, ages 65+) and sex (1 = male, 2 = female). Education level (0 = ≤ 8th grade education, 1 = 9th grade of higher) was measured as a potentially modifiable risk factor which occurs during early life. Other potentially modifiable risk factors that occur over the lifespan examined included hearing loss (0 = no, 1 = yes), hypertension (0 = no, 1 = yes), current obesity (0 = not obese, < 30.0 BMI or 1 = obese, ≤ 30.0 BMI), and current smoking (0 = no, 1 = yes). Depressive symptoms were measured by self-reported anxiety or depression (0 = no, 1 = yes) using the EQ-5D (2001–2003) and the Patient Health Questionnaire (PHQ-2) (2004–2018). Physical inactivity (0 = no, 1 = yes) was measured by doctor’s recommendation for more exercise (2001–2015) or self-report of less than 150 minutes of vigorous/moderate physical activity per week (2016–2018). Social isolation was measured by current marital status (0 = not married, 1 = married). Diabetes diagnosis was determined by asking participants if they had ever been told they had diabetes by a health care provider (0 = no, 1 = yes). Health care access (0 = none, 1 = any usual source of care) and health insurance coverage (1 = any private, 2 = public only, 3 = none) were evaluated as potential mediators that may impact ADRD diagnosis.

### Statistical Analysis

Weighted column percentages and standard errors were used to describe sample characteristics of US-born Whites, foreign-born Whites from Europe, and foreign-born MENA adults. Chi square tests were used to examine differences for all covariates. For two-group comparisons, post-hoc chi square analyses were conducted. Among all adults, age- and sex-adjusted prevalence estimates of suspected undiagnosed ADRD were calculated for all groups. Age-adjusted and sex-stratified results were also calculated for all groups.

Logistic regression procedures were used to determine associations between each region of birth among Whites (US, Europe, MENA) before and after controlling for risk factors. US-born Whites were treated as the reference group based on their historical classification as the majority group and previous research on MENA health (22). Each covariate was included in the regression models during the model building process. Model 2 included age and sex as demographic risk factors. Model 3 included education as a potentially modifiable risk for dementia during early life. In model 4, other potentially modifiable risk factors were evaluated (hearing loss, hypertension, obesity, smoking, depressive symptoms, social isolation, physical inactivity and diabetes). Statistically significant risk factors (*P*< .05) were included in multivariable models. Ultimately, model 2 adjusted for age and sex, model 3 further adjusted for education, and model 4 further adjusted for depressive symptoms. Usual source of care and health insurance were also evaluated as potential mediators; however, they were not included in the final models (p > .05). Data were analyzed using STATA 17.2 (SVYSET procedures). We conducted a sensitivity analysis limiting the sample to 2000–2014 NHIS and 2001–2015 MEPS data to account for changes in ICD-9 and ICD-10 classification codes.

The study was approved by the Agency for Healthcare Research and Quality. Data were analyzed at a local Federal Statistical Research Data Center. The study was deemed not subject to review or approval by the institutional review board because it used de-identified secondary data that does not meet the federal definition for human subjects’ research.

## Results

### Descriptive Results

Descriptive characteristics are presented in Table 1. Among all older adults, when all groups were compared, statistically significant differences were found by age, education, hearing loss, depressive symptoms, and health insurance coverage (*P*< .05). When foreign-born MENA adults were compared to foreign-born Whites from Europe, they were less likely to be female (48.5% vs. 58.6%) and younger (mean age 73.6 years vs. 75.2 years). Foreign-born MENA adults were more likely to be depressed (37.3%) compared to US- (25.7%) and foreign-born Whites from Europe (33.5%). More foreign-born MENA adults (2.2%) did not have any health insurance coverage compared to other groups (US-born Whites = 0.2%; foreign-born Whites from Europe = 0.7%). MENA men were younger (mean age 70 years vs. 74.8 years) and less likely to have lower levels of education (3.4% vs. 10.2%) compared to foreign-born Whites (*P* < .05). MENA men were less likely to be married (18.5%) than both US- (27.6%) and foreign-born (27.8%) Whites. MENA women were more likely to have lower education (18.65) than US-born White women (5.1 %). MENA women were also more likely to report depressive symptoms (40.8%) and no usual source of care (14.5%) than US-born White women (26.8% and 6.4%, respectively). Fewer MENA women had private health insurance coverage (34.9%) compared to 57.9% of US-born White women. Among foreign-born women, 79.7% of MENA women lived in the US for 15 years or longer compared to 91.5% of White women born in Europe (*P*< .05).

### Prevalence Estimates

Prevalence estimates of suspected undiagnosed ADRD are presented in Table 2. Suspected undiagnosed ADRD prevalence estimates were highest among foreign-born MENA adults (15.8%) compared to US- (8.1%) and foreign-born (11.8%) Whites from Europe. When stratified by sex, the age-adjusted prevalence of suspected undiagnosed ADRD was highest among foreign-born MENA women (22.5%) and lowest among US-born White (7.6%) men.

### Logistic Regression Results

Logistic regression results are presented in Table 3. Among adults, foreign-born MENA individuals had 1.93 times higher odds (95% CI = 1.09–3.40) of having suspected undiagnosed ADRD compared to US-born Whites in the unadjusted analysis. Results remained statistically significant after adjusting for demographics and education. MENA adults had 2.03 times greater odds (95% CI = 1.15–3.58) of suspected undiagnosed ADRD compared to US-born Whites after adjusting for age, sex, and education. Results were attenuated and no longer significant after adjusting for depressive symptoms (OR = 1.75; 95% CI = 0.97–3.18). There were no statistically significant differences between foreign-born MENA men and US-born White men. Among women, foreign-born MENA women had 2.85 times higher odds (95% CI = 1.50–5.40) of suspected undiagnosed ADRD compared to US-born Whites. Results remained statistically significant after adjusting for age and sex (OR = 3.08; 95% CI = 1.68–5.69) and education (OR = 2.77; 95% CI = 1.46–5.25). After further adjusting for depressive symptoms, foreign-born MENA women had 2.52 times greater odds (95% CI = 1.31–4.84) of suspected undiagnosed ADRD compared to US-born White women.

A different pattern was observed when foreign-born Whites from Europe were compared to US-born Whites. Among all adults, foreign-born White individuals from Europe had 1.64 times higher odds (95% CI = 1.27–2.11) of having suspected undiagnosed ADRD compared to US-born Whites. Results were attenuated and no longer significant in further models. There were no statistically significant differences between foreign-born White men from Europe and US-born White men. Foreign-born White women from Europe had 1.70 times higher odds (95% CI = 1.25–2.31) of suspected undiagnosed ADRD compared to US-born White women. Results remained statistically significant after adjusting for demographics (OR = 1.60; 95% CI = 1.17–2.19) and education (OR = 1.47; 95% CI = 1.06–2.02). After further adjusting for depressive symptoms, foreign-born White women from Europe had 1.50 times greater odds (95% CI = 1.09–2.06) of suspected undiagnosed ADRD compared to US-born White women.

### Sensitivity Analysis

Results from our sensitivity analysis are presented in Supplementary Table 1. Findings were similar to our primary results when we limited our analysis to individuals with ICD-9 classification codes (MEPS 2001 – 2015).

## Discussion

This study evaluated the prevalence of suspected undiagnosed ADRD among foreign-born MENA adults compared to US- and foreign-born Whites from Europe and compared the odds of suspected undiagnosed ADRD before and after adjusting for covariates.

Prevalence estimates were higher among foreign-born MENA adults compared to US-born Whites. MENA adults had higher odds of having suspected undiagnosed ADRD; however, sex-stratified results were only significant among women. An overview of sex-specific differences and the implications that education and depressive symptoms have as potentially modifiable risk factors for ADRD among MENA adults are discussed below.

The magnitude of the higher odds of suspected undiagnosed ADRD among MENA older adults and MENA women was attenuated when education was taken into account. Previous studies have shown that adults with undiagnosed ADRD are more likely to have lower levels of education (2). Less education is not only a risk for ADRD, but a key factor that drives underdiagnosis. Older adults with lower levels of education are less likely to receive preventive health care (23), which limits their ability to be screened during primary care visits if signs and symptoms of cognitive impairment are recognized by the healthcare provider (24). Since this is the first study to explore undiagnosed ADRD among MENA adults, we are unable to compare our results to other studies that accounted for education as a risk factor for ADRD underdiagnosis. However, our finding that education levels were lower among MENA adults compared to the US-born Whites in our sample is consistent with previous research. Studies using national data sources have reported low levels of education among foreign-born MENA adults ranging from 18–32.8% (11, 13, 25–31). When stratified by sex, studies showed that 22.7–26.7% of foreign-born MENA women (13, 32) and 11 % of foreign-born MENA men had less than high school education (13, 33). In the current study, we found that 10.5% of foreign-born MENA adults had less than a ninth-grade level of education in our bivariate analysis, which was not statistically significantly different than US- (5.9%) or foreign-born Whites (14.0%). Yet, we found a large disparity in education level when we stratified by sex. Only 3.4% of MENA men had less than a ninth-grade education compared to 6.8% of US- and 10.2% of foreign-born White men (*P*= .0385). Among women, 18.6% of MENA women had less than ninth-grade level of education compared to only 5.1% of US-born and 16.6% of foreign-born White women (p < .0001). Our results show a similar educational pattern as community-based studies in Michigan with large concentrations of older MENA/Arab populations, which found that the average years of formal education among MENA women was 2 years, with 68% reporting no formal education (34).

Depressive symptoms during late life (ages 65 and older) have also been identified as a potentially modifiable risk factor for ADRD (35). Yet, it remains unclear whether depressive symptoms are an independent risk factor for ADRD or a prodromal symptom. Self-reported cognitive limitations could reflect symptoms of depression rather than a dementia syndrome. Despite this limitation, we still found higher odds of suspected undiagnosed ADRD among MENA women even after controlling for depressive symptoms. Although depression has not been explicitly linked to ADRD underdiagnosis, previous studies have shown that older adults are less likely to receive mental health services (36) and may prefer to receive treatment from their primary care provider (37). With most older adults having other comorbid chronic conditions that need to be addressed during primary care visits (38), there may be limited opportunities for early detection and screening for ADRD while addressing other health concerns (39). Existing literature has identified a wide range (5.5%-60%) of prevalence estimates for depressive symptoms among MENA individuals using community-based convenience samples (40–42), electronic health record data (43, 44), and nationally representative samples (10, 45). For example, using linked NHIS and MEPS data, Kindratt and colleagues reported that 38.2% of MENA adults ages 65 and older had depressive symptoms (10). The prevalence was slightly higher than what we found in the current study (37.3%) that was limited to adults ages 65 and older without an ADRD diagnosis. In the current study, we found that older MENA women had a higher prevalence of depressive symptoms than men (40.8% and 34.0%, respectively). This finding is consistent with other community-based studies that demonstrate depressive symptoms were higher among MENA women compared to MENA men (34, 41, 45).

### Strengths and Limitations

A strength was the use of two nationally representative data sources to uncover MENA health outcomes while no ethnic identifier is available on a national scale. The NHIS is the only nationally representative health survey that allows for MENA individuals to be disaggregated from other foreign-born adults. Several studies have used NHIS data to uncover health outcomes among foreign-born Middle Eastern and Arab American immigrant populations (11, 13, 25, 26, 29–33). By linking with MEPS, we accessed additional health information from a subsample of participants who completed the previous year’s NHIS. To our knowledge, this methodology has only been used once for measuring MENA health. This study replicates methods used by Kindratt and colleagues (2022) to link NHIS and MEPS data and broaden the sample to be inclusive of MENA individuals by also including White Africans (10). We added MEPS medical condition data files to evaluate ADRD underdiagnosis. Although our sample size included individuals with both ICD-9 and ICD-10 codes to determine ADRD diagnosis, a sensitivity analysis was conducted to ensure our findings were not biased by the change in classification system. Removing individuals with and an ADRD diagnosis from the sample allowed us to gain a better understanding of individuals suspected to be undiagnosed.

Despite the strengths, various limitations may have affected our results. The first limitation was survey language. Both NHIS and MEPS are only collected in English and Spanish; therefore, MENA adults who only speak Arabic may have been excluded from the sample. This limitation may have resulted in an underestimate of the disparity considering that monolingual Arabic speakers may be even less likely to access high-quality medical care and be diagnosed. However, because data are collected from one key representative from each household, responses from Arabic speakers may have been given by a bilingual family member. Another limitation is our inability to disaggregate US-born MENA individuals from other US-born Whites. We may have found an even larger disparity if we were able to make these comparisons without accounting for nativity status. The cross-sectional nature of the NHIS and MEPS are a limitation in that causal links could not be ascertained. Finally, since the sample was limited to adults who reported a medical condition that was directly linked to ICD-9 or ICD-10 billing code, individuals may not have shared this information during the interview. MEPS medical condition codes were designed to measure medical expenditures, not national prevalence estimates. Despite this limitation, using MEPS medical condition data allows us to expand research on MENA health while no ethnic identifier is available.

## Conclusions

This is one of the first studies to evaluate suspected undiagnosed ADRD among MENA adults. While no other studies have examined undiagnosed ADRD among MENA adults, our findings are consistent with recent research reporting that Arab and MENA adults have a higher prevalence of cognitive limitations than other Whites (10–12). Furthermore, our findings are similar to previous studies which have shown that Black and Hispanic adults have greater risk of ADRD underdiagnosis compared to Whites (2, 3). By including MENA Americans with other Whites, it not only masks their own health outcomes, but may lessen the true disparity between privileged and other marginalized groups (6). Our results highlight that MENA health outcomes are more similar to other racial and ethnic minority groups than Whites. Without an ethnic identifier for Arab/MENA adults, cognitive health outcomes among this group will remain unnoticed and planning for future care and improving quality of life among MENA adults with suspected undiagnosed ADRD will continue to be overlooked.

## Figures and Tables

**Table 1:** Selected characteristics of US- and foreign-born adults ages 65 and older without diagnosed ADRD, 2000–2017 NHIS and 2001–2018 MEPS, n = 23,981

	US-Born	Foreign-Born		Group differences*
	NH White n = 23,069 % *(SE)*	NH White n = 774 % *(SE)*	MENA N = 138 % *(SE)*	
Demographic Factors				
Female Sex	54.9 (0.00)	58.6 (0.02)	48.5 (0.04)	USW = MENA < FBW
Age *Mean (SE)*	74.0 (0.01)	75.2 (0.30)	73.6 (0.51)	USW = MENA < FBW
Potentially Modifiable Risk Factors				
<9th grade education	5.9 (0.00)	14.0 (0.01)	10.5 (0.03)	USW = MENA = FBW
Hearing Loss (%yes)^†^	21.0 (0.00)	17.4 (0.02)	13.3 (0.04)	USW = MENA = FBW
Hypertension (%yes)	62.5 (0.00)	66.6 (0.02)	62.1 (0.05)	USW = MENA = FBW
Obesity (%yes)^‡^	25.9 (0.00)	21.5 (0.02)	25.7 (0.06)	USW = MENA = FBW
Current Smoker (%yes)^§^	9.9 (0.00)	7.5 (0.01)	8.4 (0.03)	USW = MENA = FBW
Depressive Symptoms (%yes)^¶^	25.7 (0.00)	33.5 (0.02)	37.3 (0.06)	USW < MENA = FBW*
Social Isolation (%not married)^#^	42.0 (0.01)	43.2 (0.03)	35.4 (0.06)	USW = MENA = FBW
Physical Inactivity (%yes)**	47.6 (0.01)	46.4 (0.03)	41.4 (0.08)	USW = MENA = FBW
Diabetes (%yes)	18.5 (0.00)	17.6 (0.02)	22.3 (0.05)	USW = MENA = FBW
Health Care Factors				
No usual source of care	6.6 (0.00)	6.2 (0.01)	8.7 (0.03)	USW = MENA = FBW
Health Insurance				
Any private	60.2 (0.01)	48.2 (0.02)	44.4 (0.07)	USW > MENA = FBW*
Public only	39.6 (0.01)	51.1 (0.02)	53.4 (0.07)	USW < MENA = FBW*
None	0.2 (0.00)	0.7 (0.00)	2.2 (0.01)	USW < MENA = FBW*
Acculturation Factors				
US Citizen (%yes)	–	80.9 (0.02)	81.5 (0.06)	MENA = FBW
15 + years living in US	–	91.7 (0.02)	81.5 (0.06)	MENA < FBW*
Males				
Age *Mean (SE)*	73.6 (0.01)	74.8 (0.48)	73.0 (0.77)	USW = MENA < FBW*
Potentially Modifiable Risk Factors				
<9th grade education	6.8 (0.00)	10.2 (0.02)	3.4 (0.02)	USW = MENA < FBW*
Hearing Loss (%yes)^†^	27.1 (0.01)	20.5 (0.03)	15.2 (0.06)	USW = MENA = FBW
Hypertension (%yes)	62.6 (0.01)	67.8 (0.03)	59.6 (0.08)	USW = MENA = FBW
Obesity (%yes)^‡^	26.4 (0.01)	21.6 (0.03)	30.0 (0.09)	USW = MENA = FBW
Current Smoker (%yes)^§^	11.2 (0.00)	9.8 (0.02)	10.0 (0.04)	USW = MENA = FBW
Depressive Symptoms (%yes)^¶^	24.4 (0.00)	34.2 (0.03)	34.0 (0.07)	USW = MENA = FBW
Social Isolation (%not married)^#^	27.6 (0.01)	27.8 (0.03)	18.5 (0.05)	USW > MENA < FBW*
Physical Inactivity (%yes)**	48.8 (0.01)	48.4 (0.04)	36.7 (0.10)	USW = MENA = FBW
Diabetes (%yes)	20.8 (0.01)	24.2 (0.03)	27.8 (0.08)	USW = MENA = FBW
Health Care Factors				
No usual source of care	6.8 (0.00)	5.8 (0.01)	3.2 (0.02)	USW = MENA = FBW
Health Insurance				
Any private	63.0 (0.01)	54.8 (0.04)	53.4 (0.08)	USW > MENA = FBW*
Public only	36.8 (0.01)	45.0 (0.04)	44.7 (0.08)	USW < MENA = FBW*
None	0.2 (0.00)	0.2 (0.00)	2.0 (0.02)	USW < MENA = FBW*
Acculturation Factors				
US Citizen (%yes)	–	82.7 (0.02)	86.7 (0.06)	MENA = FBW
15 + years living in US	–	91.9 (0.02)	83.2 (0.07)	MENA = FBW
Females				
Age *Mean (SE)*	74.5 (0.01)	75.4 (0.31)	74.3 (0.77)	USW = MENA = FBW
Potentially Modifiable Risk Factors				
<9th grade education	5.1 (0.00)	16.6 (0.02)	18.6 (0.05)	USW< MENA = FBW*
Hearing Loss (%yes)^†^	16.0 (0.00)	15.2 (0.02)	11.3 (0.04)	USW = MENA = FBW
Hypertension (%yes)	62.4 (0.01)	65.8 (0.03)	64.7 (0.07)	USW = MENA = FBW
Obesity (%yes)^‡^	25.4 (0.01)	21.4 (0.02)	21.3 (0.06)	USW = MENA = FBW
Current Smoker (%yes)^§^	8.8 (0.00)	5.8 (0.01)	6.6 (0.03)	USW = MENA = FBW
Depressive Symptoms (%yes)^¶^	26.8 (0.00)	33.1 (0.03)	40.8 (0.08)	USW < MENA = FBW*
Social Isolation (%not married)^#^	53.7 (0.01)	54.1 (0.03)	53.3 (0.08)	USW = MENA = FBW
Physical Inactivity (%yes)**	46.5 (0.01)	44.9 (0.03)	46.3 (0.10)	USW = MENA = FBW
Diabetes (%yes)	16.6 (0.00)	12.9 (0.02)	16.5 (0.05)	USW = MENA = FBW
Health Care Factors				
No usual source of care	6.4 (0.00)	6.4 (0.01)	14.5 (0.05)	USW < MENA > FBW*
Health Insurance				
Any private	57.9 (0.01)	43.6 (0.03)	34.9 (0.08)	USW > MENA = FBW*
Public only	42.0 (0.01)	55.4 (0.03)	62.7 (0.08)	USW < MENA = FBW*
None	0.2 (0.00)	1.0 (0.01)	2.4 (0.02)	USW < MENA = FBW*
Acculturation Factors				
US Citizen (%yes)	–	79.8 (0.02)	76.0 (0.07)	MENA = FBW
15 + years living in US	–	91.5 (0.02)	79.7 (0.07)	MENA < FBW*

Abbreviations. FBW = Foreign-born non-Hispanic White; MENA = Middle Eastern or North African; MEPS = Medical Expenditure Panel Survey; NH = non-Hispanic; USW = US-born non-Hispanic White.

* p < .05, chi square test for comparisons between US-born non-Hispanic Whites, foreign-born non-Hispanic Whites from Europe/Russia, and foreign-born MENA Americans.

† Hearing loss (yes or no) was determined by self-report of any hearing difficulty, including some or serious difficulty.

‡ Obesity was determined by self-reported body mass index (BMI) of ≥ 30 kg/m^2^ (yes or no).

§ Current smokers were compared to former and never smokers (“no” responses)

¶ Depressive Symptoms were determined by self-report of problems with anxiety or depression as measured by EQ-5D (2001 – 2003) or score of 2 or greater on Patient Health Questionnaire (PHQ2) (2004–2018) measuring little interest or pleasure or feeling down/depressed.

# Social isolation determined by current marital status (yes or no). “No” responses included divorced, widowed, and separated responses.

** Physical inactivity (yes or no) determined by receipt of doctor’s advised to exercise more (2001 – 2015) or self-report or current moderate to vigorous physical activity at least one half-hour five times a week.

**Table 2:** Prevalence estimates of suspected undiagnosed ADRD among US- and foreign-born adults ages 65 and older,* 2000–2017 NHIS/2001 – 2018 MEPS, n = 23.981.

	US-Born	Foreign-Born	
	NH White %*(SE)*	NH White % *(SE)*	MENA % *(SE)*
*Age- and sex-adjusted*			
Suspected Undiagnosed ADRD	8.1 (0.00)	11.8 (0.01)	15.8 (0.04)
*Age-adjusted and sex-stratified*			
Males^†^			
Suspected Undiagnosed ADRD	7.6 (0.00)	10.3 (0.02)	9.3 (0.04)
Females^‡^			
Suspected Undiagnosed ADRD	8.6 (0.00)	13.1 (0.02)	22.5 (0.05)

Abbreviations. ADRD = Alzheimer’s disease and related dementias; MENA = Middle Eastern or North African; MEPS = Medical Expenditure Panel Survey; NH = non-Hispanic.

* Total N = 23,981 (n = 23,069 for US-born non-Hispanic (NH) white; n = 774 for foreign-born NH white; n = 138 for foreign-born Middle Eastern or North African (MENA).

† Total Males N = 10,766 (n = 10,380 for US-born NH white; n =317 for foreign-born NH white; n = 69 for foreign-born MENA.

‡ Total Females N = 13,215 (n = 12,689 for US-born NH white; n = 457 for foreign-born NH white; n = 69 for foreign-born MENA.

**Table 3:** Crude and adjusted logistic regression models for suspected undiagnosed ADRD, ages 65 and older, * 2000–2017 NHIS/2001 –2018 MEPS,* n = 23,981.

	Model 1^§^	Model 2^¶^	Model 3^#^	Model 4**
	OR (95% CI)	OR (95% CI)	OR (95% CI)	OR (95% CI)
US-born NH white	1.00	1.00	1.00	1.00
Foreign-born NH white	1.64 (1.27, 2.11)	1.52 (1.17, 1.97)	1.41 (1.07, 1.85)	1.32 (1.01, 1.74)
Foreign-born MENA	1.93 (1.09, 3.40)	2.12 (1.22, 3.71)	2.03 (1.15, 3.58)	1.75 (0.97, 3.18)
*Males* ^†^				
US-born NH white	1.00	1.00	1.00	1.00
Foreign-born NH white	1.52 (1.03, 2.24)	1.40 (0.93, 2.08)	1.35 (0.90, 2.05)	1.20 (0.78, 1.84)
Foreign-born MENA	1.14 (0.47, 2.77)	1.25 (0.50, 3.11)	1.37 (0.55, 3.41)	1.10 (0.42, 2.91)
*Females* ^‡^				
US-born NH white	1.00	1.00	1.00	1.00
Foreign-born NH white	1.70 (1.25, 2.31)	1.60 (1.17, 2.19)	1.47 (1.06, 2.02)	1.50 (1.09, 2.06)
Foreign-born MENA	2.85 (1.50, 5.40)	3.08 (1.68, 5.69)	2.77 (1.46, 5.25)	2.52 (1.31, 4.84)

* Total N = 23.981 (n = 23,069 for US-born non-Hispanic (NH) white; n = 774 for foreign-born NH white; n = 138 for foreign-born Middle Eastern or North African (MENA).

† Total Males N = 10,766 (n = 10,380 for US-born NH white; n= 317 for foreign-born NH white; n = 69 for foreign-born MENA.

‡ Total Females N = 13,215 (n = 12,689 for US-born NH white; n = 457 for foreign-born NH white; n = 69 for foreign-born MENA.

§ Unadjusted model.

¶ Adjusted for age and sex for all adults, adjusted for age among models stratified by sex.

# Adjusted for Model 2 + eduction.

** Adjusted for Model 3 + depressive symptoms.
